# Identification of gene expression profiles and immune cell infiltration signatures between low and high tumor mutation burden groups in bladder cancer

**DOI:** 10.7150/ijms.39056

**Published:** 2020-01-01

**Authors:** Zonglong Wu, Muru Wang, Qinggang Liu, Yaxiao Liu, Kejia Zhu, Lipeng Chen, Hongda Guo, Yan Li, Benkang Shi

**Affiliations:** 1 Department of Urology, Qilu Hospital of Shandong University, Jinan, China; 2 Division of Gastroenterology, Tongji Hospital, Huazhong University of Science and Technology, Wuhan, China

**Keywords:** tumor mutation burden, bladder cancer, gene expression profile, immune cell, TCGA database

## Abstract

Bladder cancer is one of the most commonly diagnosed tumors and is results from the accumulation of somatic mutations in the DNA. Tumor mutation burden (TMB) has been associated with cancer immunotherapeutic response. In this study, we attempted to explore the correlation between TMB and cancer prognosis. Identify the different expressed genes and immune cell infiltration signatures between low and high TMB group. Mutation data, gene expression profiles and clinical data were downloaded from The Cancer Genome Atlas (TCGA) database. Patients were divided into high and low TMB groups, allowing differentially expressed genes (DEGs) to be identified. Functional enrichment and protein-protein interaction (PPI) network analysis were used to identify the functions of the DEGs. And immune cell infiltration signatures were evaluated by CIBERSORT algorithm. These results shown that high TMB was significantly associated with prognosis. We obtained a list of TMB related genes which may influence the infiltrations of immune cells. We also found a higher proportion of CD8 T cells, CD4 T cells and NK cells in the high TMB group. Our data suggest that higher TMB tends to promote the infiltrations of T cells and NK cells and patients with higher TMB may achieve a more favorable prognosis in bladder cancer.

## Introduction

Bladder cancer (BLCA) is the most common carcinoma in the urinary system and remains a major global medical problem despite the numerous new treatment options available, BLCA has a high recurrence rate which become a major economic burden on the health care systems 1, 2. The cause of BLCA is unclear. Smoking, environmental factors, exposure to toxic industrial chemicals and gases, bladder inflammation are thought to be associated with bladder cancer 3. Despite improvements in clinical outcomes in recent years, there are still many issues to be aware of, such as patients with invasive tumors or metastatic have short survival times. Recently, immunotherapy target the programmed cell death 1 programmed cell death 1 (PD-1), PD-1 ligand (PD-L1), cytotoxic T-lymphocyte antigen-4 (CTLA-4) offers great promise for various cancer therapies 4-6.

Many studies have explored the link between immunotherapy response and tumor mutation burden (TMB) 7-10. Mutations in tumor cells can be transcribed and translated, and may form new antigens can be identified and targeted by the immune system 11, 12. In fact, not all mutations will generate immunogenic, only a few mutations can be recognized by T cells 13-15. The more tumor mutations, the more antigens it may form. Higher TMB tends to form more new antigens, making tumors more immunogenic and improving clinical response to immunotherapy 9. So TMB can be used to estimate the new antigen load of a tumor.

The Cancer Genome Atlas (TCGA) has been established to map the genome variations of human cancers using genomic analysis techniques, providing a wealth of mutation and expression profile data. In this study, we identified different expression genes (DEGs) between high and low TMB groups and evaluate the relationship of immune cell infiltration signatures and TMB by using the information of patients with bladder cancer from the TCGA database.

## Materials and Methods

### Database and genomic analysis

The mutation data, gene expression profiles and clinical data of patients with bladder cancer were obtained from the TCGA data portal (https://tcga-data.nci.nih.gov/tcga/) and the maftools package was used to analyze and summarized the mutation data. TMB was calculated from the tumor specific mutation genes. Gene expression data analysis was performed using the R software package, limma. A fold change of > 1.5 and false discovery rate (FDR) of < 0.05 were used as cutoffs to identify DEGs. Volcano plots and heat maps were generated using the ggplot2 and pheatmap packages respectively.

### DEGs enrichment analysis and protein-protein interaction (PPI) network construction

Gene ontology (GO) enrichment analysis and Kyoto Encyclopedia of Genes and Genomes (KEGG) pathway enrichment analysis were performed using the clusterProfiler package 16. The STRING database 17 and cytoscape software 18 were used to retrieve and reconstruct a PPI network. Important nodes and subnetworks were predicted and explored using cytohubba, a cytoscape plugin 19, and the top 10 hub genes were selected from the results of each method.

### Gene set enrichment analysis (GSEA) analysis

GSEA was used to further understand CXCL10-related pathways. The expression level of CXCL10 was used as the phenotype label and bladder cancer patients in the TCGA cohort were divided into two groups based on the median expression value of CXCL10. The collection of annotated gene sets of c2.cp.kegg.v6.2.symbols.gmt was chosen as the reference gene sets and GSEA version 3.0 software was used to analyze our data. FDR < 0.01 was used as the cut-off criteria.

### Evaluation of tumor-infiltrating immune cells

CIBERSORT algorithm was used to calculate the fractions of infiltrating immune cells. CIBERSORT is an analytical tool that estimates specific cell types in a mixed cell population using gene expression data. And the algorithm was run using the 1000 permutations and LM22 signature 20. CIBERSORT method was used to quantify the fractions of immune cells in the bladder cancer samples. At a threshold of P < 0.05, the results of the inferred fractions of immune cell populations produced by CIBERSORT were considered accurate 20.

### Statistical analysis

We divided patients into the high TMB or the low TMB groups with the median of mutation frequency as the threshold value. Kaplan‐Meier survival curves were obtained and compared by log‐rank tests. The associations of clinicopathologic characteristics and corresponding TMB were analyzed by one-way ANOVA followed by Tukey's multiple-comparison post-hoc test and unpaired two-tailed t test. The difference of infiltrating immune cells between high TMB group and low TMB group was assessed using the unpaired t test.

## Results

### Primary genetic alterations in bladder cancer patients

**Figure 1:**
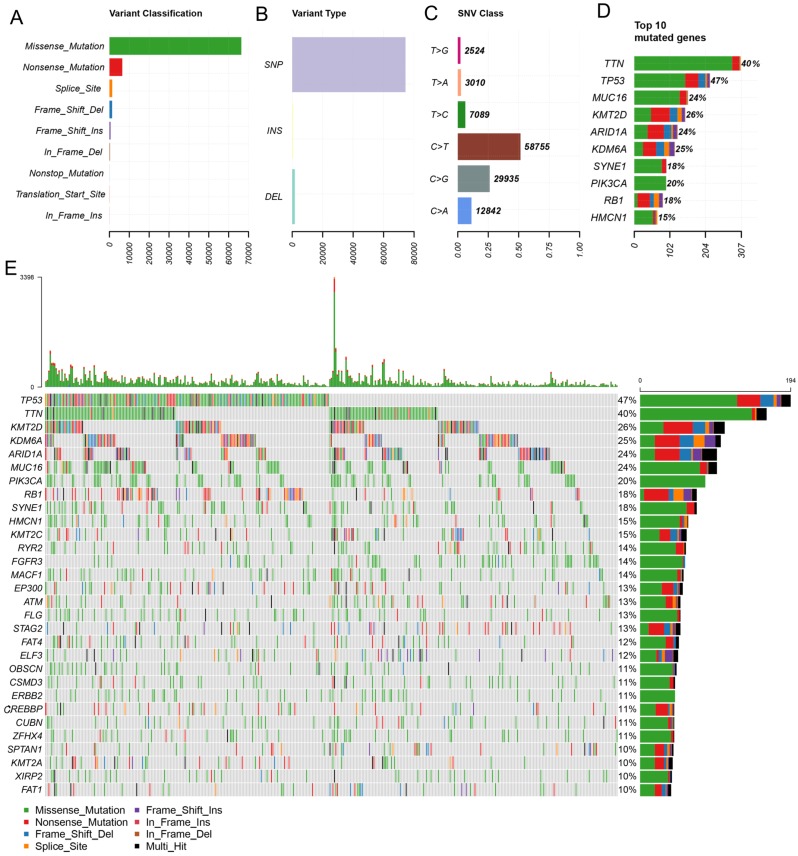
** Primary genetic alterations in bladder cancer patients.** (A, B) Variant classification and type of genetic alterations in bladder cancer. (C). The SNV class of bladder cancer. (D, E) Top 10 mutant genes and mutation profile of bladder cancer.

In this study, the clinical information and the results of whole-exome sequencing of patients with BLCA were downloaded from the TCGA database. By using maftools, mutation data were analyzed and summarized. The mutations were further classified according to the variant effect predictor, among these mutations, missense mutations are the most common (Figure 1A). And the most common mutations type is SNP (Figure 1B). C > T transversion is the most common type of SNV in bladder cancer (Figure 1C). And the top 10 mutated genes are TTN, TP53, MUC16, KMT2D, ARID1A, KDM6A, SYNE1, PIK3CA, RB1, HMCN1(Figure 1D, E).

### Correlation of TMB with prognosis, clinicopathological characteristics and tumor grades of BLCA patients

**Figure 2:**
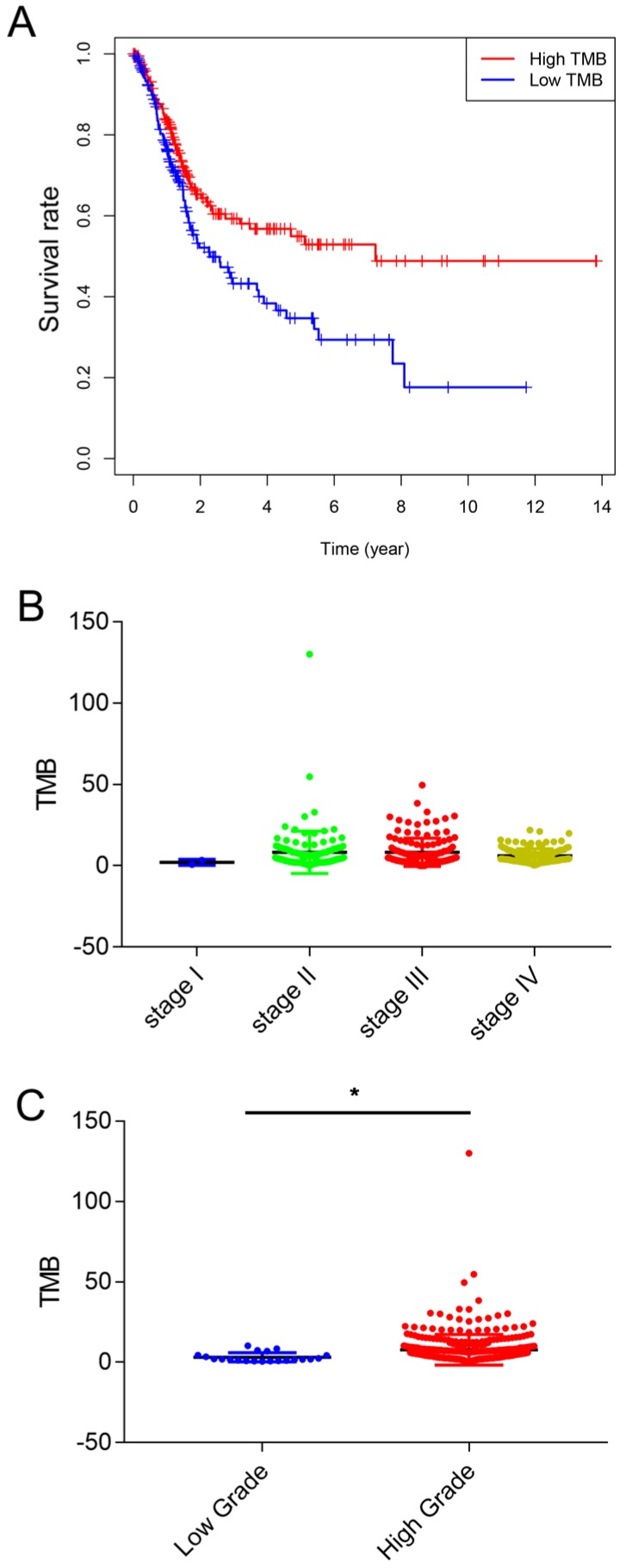
** Correlation of TMB with prognosis, clinicopathological characteristics and tumor grades of BLCA patients.** (A) Patients with BLCA were divided into two groups based on their TMB. As shown in the Kaplan‐ Meier survival curve, patients with high-TMB had a higher overall survival than those with low-TMB (hazard ratio [HR] 1.562; 95 % CI 1.14-2.14; P= 0.005 by log-rank test). (B) The TMB showed no statistically significant differences at different pathological stages (by one-way ANOVA followed by Tukey's multiple-comparison post-hoc test). (C) Higher TMB level correlated with advanced tumor grades (*, P<0.05; by unpaired two-tailed t test)

TMB was calculated as the number of nonsynonymous protein coding variants divided by the total sequenced genome length. Next, we divided the patients with bladder cancer into high and low TMB groups based on the median TMB. The clinicopathologic characteristics of the patients are shown in Table 1. Kaplan-Meier survival analysis revealed that patients with high TMB had a higher survival rate than those with low TMB (Figure 2A). Next, we analyzed the relationship between TMB and clinical stage, and the results showed that TMB had no relationship with the clinical stage (Figure 2B). And higher TMB level correlated with advanced tumor grades (Figure 2C).

### Comparison of the gene expression profiles of patients in different TMB groups

**Figure 3:**
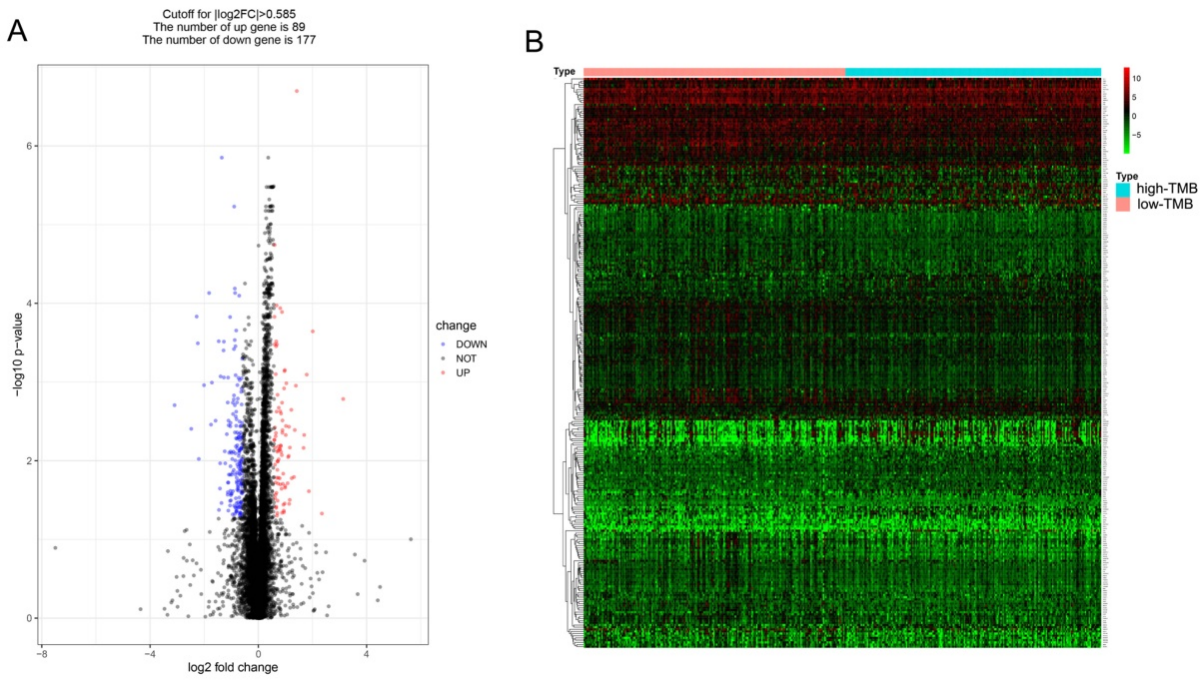
** Comparison of the gene expression profiles of patients in different TMB groups.** (A, B) The volcano plot and heatmap show the 266 genes (89 up-regulated and 177 down-regulated) identified based on the TMB.

Patients were divided into low and high TMB groups and their gene expression profiles were analyzed to identify DEGs with FDR < 0.05 and fold change of > 1.5. A total of 266 DEGs (89 up-regulated and 177 down-regulated) were identified in high TMB group (Figure 3A) and visualized using a heatmap (Figure 3B). And the list of DEGs is shown in supplementary Table S1.

### Functional enrichment and PPI network analysis of differentially expressed genes

**Figure 4:**
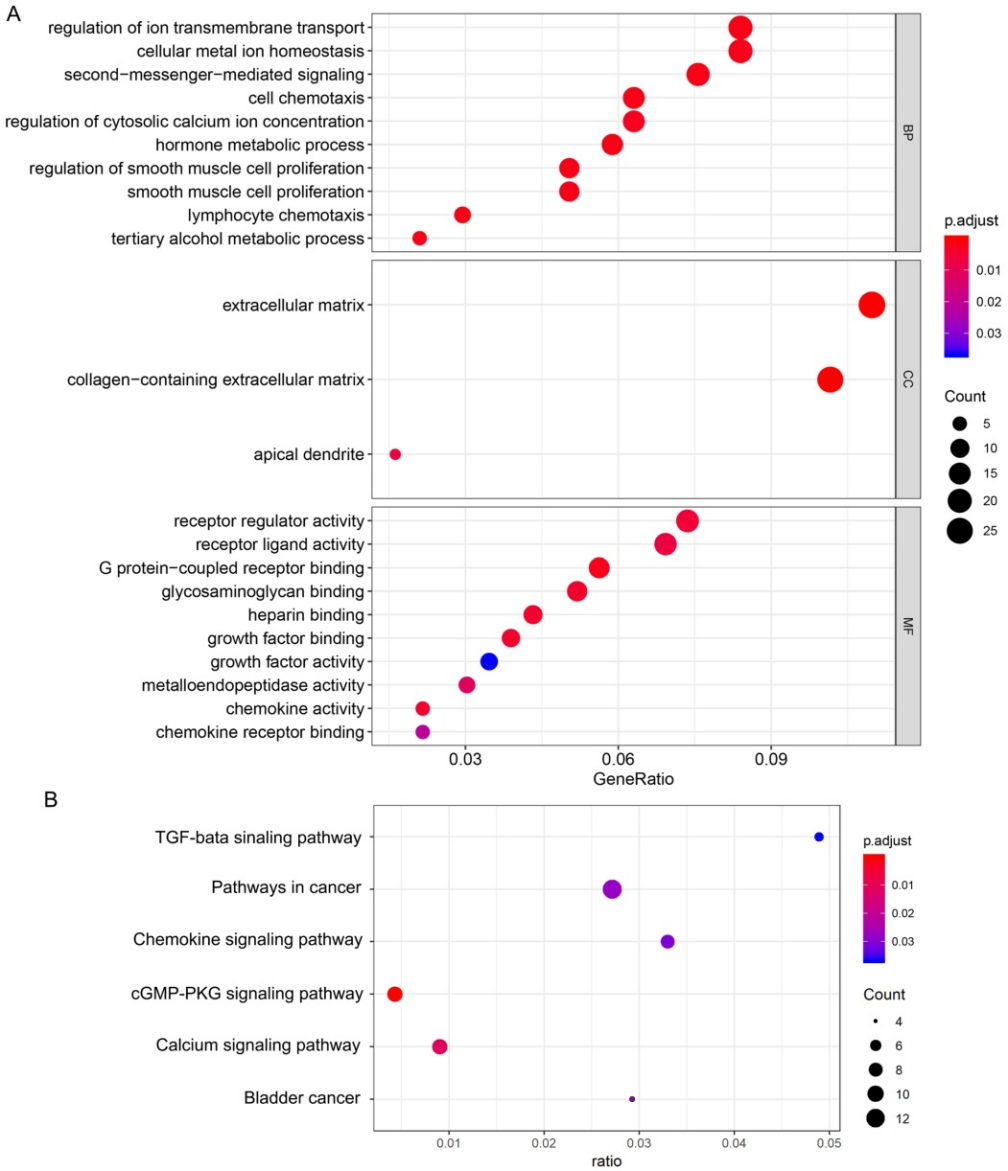
** Functional enrichment of differentially expressed genes.** (A) Biological process, cellular component, and molecular function terms for the DEGs. (B) KEGG pathways enriched for the DEGs.

**Figure 5:**
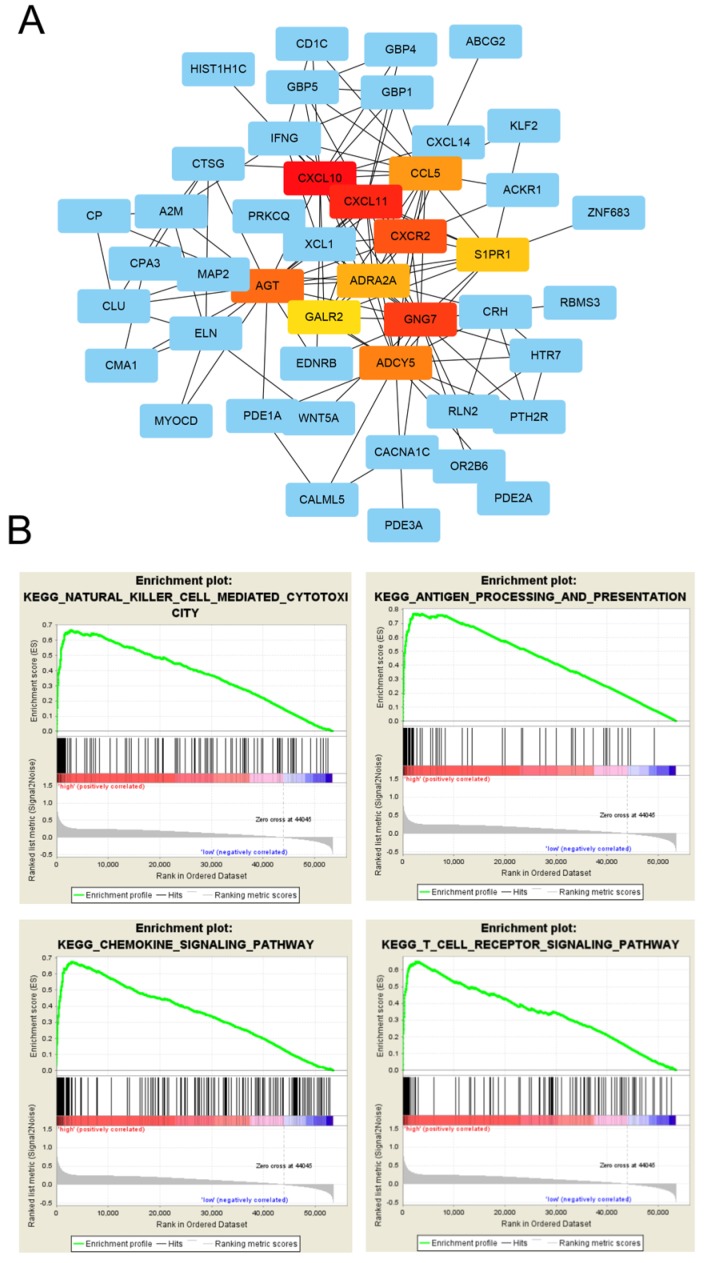
** PPI network analysis of differentially expressed genes.** (A) Protein-protein interaction networks of the DEGs. (B) CXCL10 correlated enrichment gene analysis with GSEA.

GO enrichment analysis was used to determine the functions of the 266 DEGs (Figure 4A). In BP category, “cell chemotaxis”, “lymphocyte chemotaxis” and “regulation of ion transmembrane transport” were enriched, which means the DEGs affects the consists of immune cells in tumor microenvironment. The enriched CC terms included “extracellular matrix”, “collagen-containing extracellular matrix”, “apical dendrite”, and the enriched MF terms included “chemokine activity”, “chemokine receptor binding”, “G protein-coupled receptor binding”. We also performed KEGG pathway enrichment analysis to determine the pathways most enriched for DEGs, which included “TGF-beta signaling pathway”, “chemokine signaling pathway” and “pathways in cancer” (Figure 4B). Next, we explored the relationships between the DEGs, The Search Tool for the Retrieval of Interacting Genes (STRING) database and Cytoscape software were used to construct a PPI network for the DEGs (Figure 5A). The important nodes and subnetworks of the PPI were predicted and explored using CytoHubba; the 10 most significant node genes were CXCL10, CXCL11, GNG7, CXCR2, AGT, ADCY5, CCL5, ADRA2A, S1PR1, GALR2. Next, we analyzed the pathway of high-expression samples of CXCL10 by GSEA analysis. The results showed that high CXCL10 expression samples were mainly enriched in natural killer cell mediated cytotoxicity, antigen processing and presentation, chemokine signaling pathway and T cell receptor signaling pathway (Figure 5B).

### Correlation of TMB with immune signatures in bladder cancers

**Figure 6:**
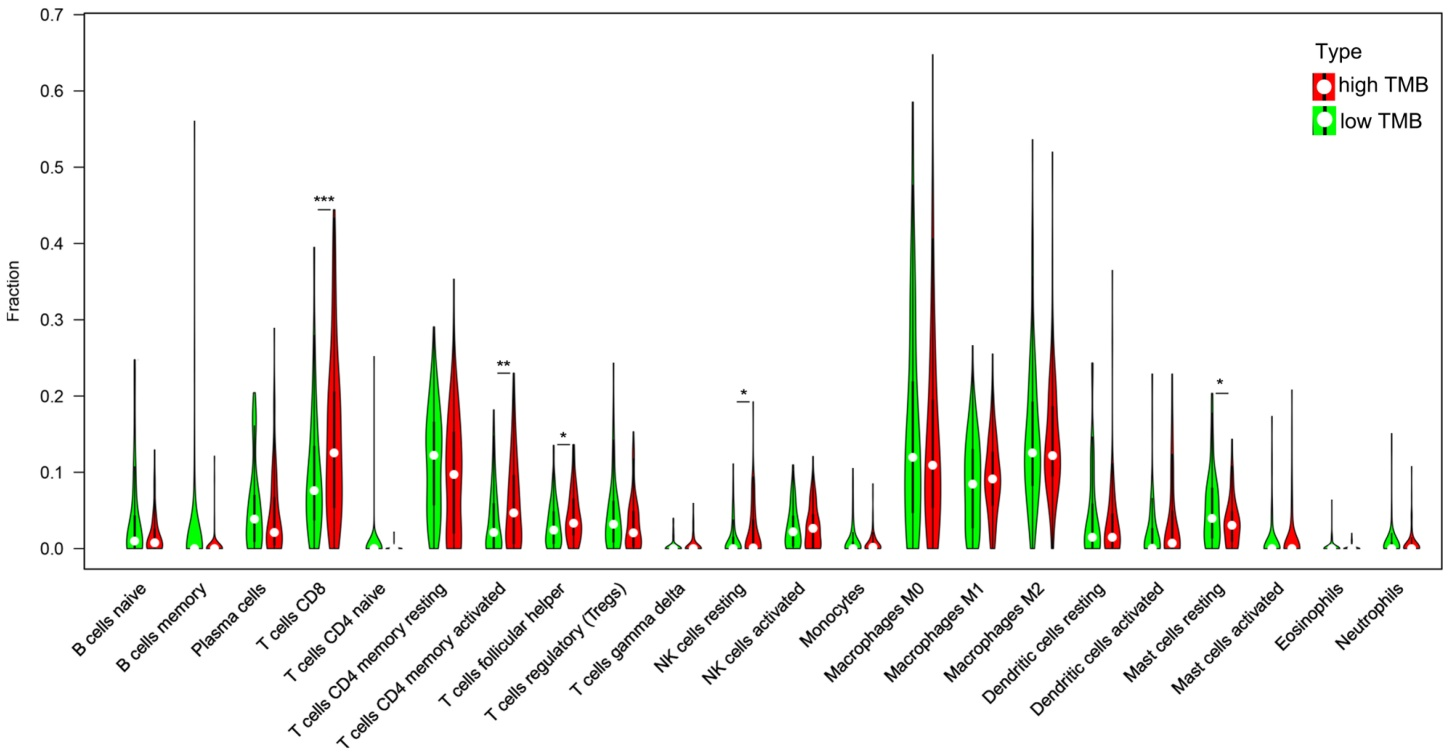
** Correlation of TMB with immune signatures in bladder cancers.** 22 types of adaptive and innate immune cells in high and low TMB groups. (*, P<0.05; **, P<0.01; ***, P<0.001; by unpaired two-tailed t test).

Previous studies have shown that the higher mutation burden in tumors tends to form more new antigens, making tumors to have higher immunogenicity 9. The GO enrichment analysis shows the DEGs involve in cell chemotaxis, so we analyzed the correlation of TMB with immune signatures in bladder cancers. CIBERSORT algorithm was used to calculated the fractions of infiltrating immune cells. At a threshold of P < 0.05, the results of the inferred fractions of immune cell populations produced by CIBERSORT were considered accurate. There are 90 patients in low TMB group and 104 patients in high TMB group with the P value < 0.05 and their inferred fractions of immune cell were considered accurate. The results shown that tumors with high TMB were significantly associated with high fractions of CD8 T cells, CD4 memory T cells, follicular helper T cells and resting NK cells. In low TMB group there is a higher fraction of mast cell. (Figure 6).

## Discussion

Cancer is a genetic disease and results from the accumulation of somatic mutations in the DNA 21. Genetic changes in tumors include nonsynonymous mutations, synonymous mutations, insertions or deletions, and copy number gains and losses. And nonsynonymous mutations mainly comprised of missense mutations (point mutations that change the amino acid codon). In different tumor types and individual tumors, there is obvious difference in the frequency of each type of these genetic alterations 22. The genetic changes increase the tumors' immunogenic. To avoid detection and killing by the host immune system, tumors often upregulate immune checkpoints. Recently, the overall survival (OS) rates of bladder cancer have increased with the therapy of immune checkpoint 23. TMB can be used to predict the efficacy of immune checkpoint blockade therapy, and can been seen as a useful biomarker to identify the patients who will benefit from immunotherapy 9, 10, 24.

High TMB cases can be seen in almost every type of cancer 25. In different tumor types, melanomas have the highest levels of TMB followed by non-small-cell lung carcinoma and other squamous carcinomas. Leukemias and pediatric tumors usually have the lowest levels of TMB 21. And TMB also has a significant difference in the same cancer type. A high TMB probably reflects the presence of mutation-associated neoantigens, with consequent increased lymphocyte infiltration in the tumor microenvironment. This phenomenon has been observed in other tumors 26, 27. The tumor microenvironment consists of immune cells, mesenchymal cells, endothelial cells, extracellular matrix (ECM) molecules, and inflammatory mediators. BLCA is an immunosensitive tumor which is infiltrated by tumor-infiltrating immune cells (TIICs) including T cells, macrophages, dendritic cells, neutrophils and mast cells 28-30. Studies have shown that the tumor microenvironment affects the gene expression of tumor tissues and the patient outcome, and therefore, has a diagnostic and prognostic value for BLCA 31. In present study, we found that TMB affects the prognosis of bladder cancer. High TMB may reflect the presence of new antigens, thereby increasing tumor-infiltrating immune cells in the tumor microenvironment which closely related to the effectiveness of targeted drugs and clinical outcomes. We also identified 266 DEGs (89 up-regulated and 177 down-regulated) between low and high TMB groups. Among the DEGs, many genes involve immune response and chemokine signaling pathway. GO term and KEGG analysis revealed that the DEGs affects the cell chemotaxis, intercellular signaling, ion transport, and the formation of extracellular matrix. These data indicated that TMB is closely related with the tumor microenvironment and these TMB-related genes cause the changes of tumor microenvironment. CXCL10 is the most significant node genes Among all DEGs and high CXCL10 expression samples were mainly enriched in natural killer cell mediated cytotoxicity, antigen processing and presentation, chemokine signaling pathway and T cell receptor signaling pathway. Chemokines and cytokines are well known to guide macrophages, T-cells and other immune cells to the tumor microenvironment and influence the outcome of the patients 32. CXCL10 may play an important role in regulating immune cell migration, differentiation, and activation in bladder cancer.

Immune-cell infiltration is a characteristic of cancer, and many cancers have a complex chemokine network that affect the extent and phenotype of this infiltrate, as well as tumor cell growth, survival and migration 33. In this study, we found high TMB group has higher fractions of CD8 T cells, CD4 T cells and NK cell and in low TMB group; there is a higher fraction of mast cell. These data indicated that TMB can affect the immune cell infiltration signatures and high TMB attracted effector cells of the immune system. Tissue resident memory T cells are a key factor in making tumors dormant; hence, it is essential to establish a cancer-immune system balance 34. Under hypoxic conditions, CD8 T cells can differentiate into lytic effector cells, increase the expression of interferon gamma (IFNγ), Fas ligand (FASL), granule B (GZMB), and inhibit tumor cell proliferation 35, 36. High infiltration of T lymphocytes in tumors is positively correlated with the survival rate of patients with bladder cancer 37. NK cell typically account for 5-15% of peripheral blood lymphocytes and respond to their targets without prior antigen sensitization 38. NK cells can recognize bladder tumor cells and their activity is important to against bladder tumor cells 39. Mast cells may contribute to tumor angiogenesis and play an important role in the growth of tumors 40. These data indicated that TMB is closely related with the immune microenvironment. Mast cells in the low TMB group may promote tumor growth and metastasis. High TMB tends to cause the chemotaxis of immune cells in BLCA and the crosstalk between these cells play an important role in the growth of tumors.

In summary, our data implicate that higher-TMB patients could gain a more favorable prognosis in bladder cancer. We also obtained a list of TMB related genes which may influence the infiltrations of immune cells. Our data provide insights into the correlation between TMB and immune cell infiltration signatures in bladder cancer and may be helpful for the exploration of the role of TMB in BLCA.

## Supplementary Material

Supplementary figures and tables.

## Figures and Tables

**Table 1:** 

		TMB		*P*-value
Variables	total patients	low	high	
**age**	68.05±10.61	67.3±11.27	68.8±9.885	
**age**				0.117
<60	87	50	37	
>=60	316	151	165	
**gender**				0.**042**
female	106	62	44	
male	297	139	158	
**grade**				0.**011**
low Grade	20	16	4	
high Grade	383	185	198	
**stage**				0.513
stage I	2	2	0	
stage II	129	63	66	
stage III	139	67	72	
stage IV	135	70	65	
