# Supplementary material for: Identification of gene expression profiles and immune cell infiltration signatures between low and high tumor mutation burden groups in bladder cancer
Source: Int J Med Sci. 2020 Jan 1;17(1):89–96. doi: 10.7150/ijms.39056 (PMC6945555; doi:10.7150/ijms.39056)
Supplement: Supplementary file 1 — Supplementary figures and tables. [file ijmsv17p0089s1.pdf]

gene	low TMB	high TMB	log2FC	FDR
GPC2	1.140292	2.549063	1.160564	0.03143
MAGEA1	2.100346	4.237477	1.012578	0.004712
ZNF683	0.830398	1.781864	1.101513	0.008998
KLF2	9.62436	6.198673	-0.63473	0.039855
GPRIN2	1.763354	2.920732	0.728008	0.007427
WFDC1	1.702049	1.125584	-0.5966	0.005007
PDE2A	1.29882	0.848221	-0.61469	0.013107
PDE1A	0.645162	0.405977	-0.66826	0.045646
CH25H	2.635447	1.524162	-0.79003	0.043433
ADAMTS1	11.15599	6.965787	-0.67946	0.014985
LSP1	23.76326	11.27007	-1.07624	0.007924
C2orf40	1.144154	0.430577	-1.40994	0.010776
IL1RL1	0.876148	0.266723	-1.71583	0.001017
IGSF9	7.216595	11.59785	0.684467	0.000332
HIST1H1C	107.3459	164.6726	0.617333	0.006804
CD1A	1.54358	0.75844	-1.02517	0.003301
VCX	0.35653	1.437088	2.011053	0.000227
GRIN2D	4.154521	6.34929	0.611913	0.003781
DAPL1	4.839127	2.223552	-1.12188	0.018947
HORMAD1	0.72576	1.426637	0.975055	0.035007
TERT	0.442781	0.821247	0.891224	0.003078
MFAP4	37.8422	21.4472	-0.81921	0.043849
SLFN13	2.173959	3.379691	0.636567	0.018883
DGCR6	0.426323	0.818614	0.941237	0.043066
GPIHBP1	0.891622	0.466135	-0.93568	0.00255
SLC25A27	1.794298	1.086646	-0.72354	0.008704
HPGDS	0.647929	0.389453	-0.73438	0.00503
CSAG2	0.553629	1.448492	1.38756	0.001114
PALD1	2.835137	1.839498	-0.62411	0.006804
MEOX1	1.203095	0.608021	-0.98456	0.015055
CSAG1	4.798457	12.19984	1.34622	0.004073
SESN3	5.308951	2.901388	-0.87168	0.000293
SCARA5	1.23318	0.447009	-1.46401	0.000304
BMP5	0.842594	0.388253	-1.11784	0.000878
MNS1	1.617185	2.527774	0.644383	0.000794
CACNA1H	2.789574	1.671013	-0.73932	0.005072
YPEL1	0.571574	0.935063	0.710123	0.010165
LRMP	6.929206	4.31969	-0.68176	0.012846
SGCA	1.958021	1.004582	-0.9628	0.009501
SMC1B	0.54559	1.000387	0.874669	0.000129
HSD17B2	7.205621	4.144526	-0.79792	0.016506
ATP1A2	1.468825	0.302656	-2.27891	0.000147
HBB	19.17728	12.06069	-0.66909	0.003651

NELL2	1.276248	2.270357	0.83101	0.006527
ADCY5	1.400314	0.874259	-0.67962	0.005447
PDGFD	3.053981	1.481524	-1.04361	0.000149
LSAMP	0.940599	0.535572	-0.8125	0.044727
GTSF1	2.090793	3.91537	0.905099	0.019671
TUBB4A	0.398602	0.651941	0.709791	0.032156
SLC14A1	20.49637	12.19436	-0.74916	0.033677
HBA2	10.72318	5.425776	-0.98283	0.007964
MSX1	3.812109	2.347162	-0.69967	0.007438
COX7B2	1.303782	4.502212	1.787931	0.000805
OSBP2	2.504369	3.887877	0.634536	0.02707
KIF26A	0.718568	0.414661	-0.79319	0.019154
RSPO4	1.080214	2.004022	0.891581	0.004089
SCN4B	0.721698	0.388748	-0.89256	5.90E-06
MAGEA10	2.410432	5.282177	1.131841	0.003702
CXCR2	0.922243	0.570249	-0.69355	0.003179
CNTD2	0.422736	0.833161	0.978839	0.000704
TIGD3	0.592675	0.938104	0.662507	0.000305
SPARCL1	44.80746	26.01743	-0.78426	0.00525
SNED1	0.633034	0.364416	-0.7967	0.018351
PTH2R	0.637673	1.245811	0.966195	0.000726
CYP39A1	1.344763	0.759843	-0.82358	0.005197
MAGEA4	6.463074	11.79473	0.86785	0.008486
CYP3A5	5.751423	2.99486	-0.94143	0.00183
KCND3	0.953937	0.583673	-0.70873	0.010165
COCH	1.411617	2.591448	0.87641	0.045847
AKR1C3	47.42441	29.9363	-0.66373	0.011228
CALML5	26.94295	137.2706	2.349043	0.046897
F10	0.770063	0.413273	-0.89788	0.001481
RHOJ	2.722524	1.736587	-0.64869	0.03389
CLDN5	4.669752	3.011543	-0.63284	0.025134
GALR2	0.553687	1.024433	0.887683	0.004414
MESP2	0.517597	0.925704	0.838722	0.002516
CCDC60	0.682995	0.403803	-0.75822	0.001624
AGT	3.607691	1.762317	-1.0336	0.003651
3-Sep	1.300975	2.266264	0.800722	0.007167
RGS5	9.528328	5.508691	-0.79051	0.014123
HES6	3.895761	12.40497	1.670941	0.006877
RBMS3	0.917711	0.535228	-0.77789	0.011134
PRAME	3.430302	5.769367	0.750077	0.009599
IQCA1	1.029358	0.619489	-0.73259	0.000887
BMX	1.235529	0.269002	-2.19944	0.009491
IGSF11	0.543709	0.820008	0.592802	0.005484
SPON1	8.050776	4.212429	-0.93448	0.024331

LRRN4CL	1.261052	0.781073	-0.6911	0.010915
GATA5	0.687287	0.314479	-1.12795	0.025134
MYOCD	0.698323	0.345554	-1.01499	0.027431
GBP4	9.010091	14.67082	0.703336	0.013069
STON1	1.578129	1.051639	-0.58557	0.009609
PLCH2	5.302579	3.008101	-0.81784	0.002381
PRKCQ	1.391078	0.886521	-0.64998	0.037776
A2M	93.94278	54.80089	-0.77758	0.006974
PHYHIP	1.401378	0.649758	-1.10887	0.018947
CDKN2A	11.79941	19.65224	0.735979	0.006603
PTPRN2	1.421884	0.778128	-0.86972	0.001969
CD207	1.840533	1.044029	-0.81796	0.000222
DYNC2H1	1.036348	0.638283	-0.69924	0.007476
BMP8B	2.245009	3.43609	0.614047	0.011225
ADAMTS8	0.780794	0.320137	-1.28625	0.000397
CSAG3	1.21284	2.461179	1.02096	0.003622
SLC16A4	1.537435	0.912803	-0.75215	0.02048
ANXA10	27.24447	11.51739	-1.24215	0.007597
FOXL1	2.663116	1.488097	-0.83965	0.000819
CLEC3B	3.288684	2.018637	-0.70413	0.014558
RLN2	0.414458	0.626632	0.596393	0.000148
LRRC4B	0.918626	0.533175	-0.78487	0.004375
DMBX1	1.403507	2.78557	0.988936	0.020989
FCMR	4.355985	2.020395	-1.10836	0.043001
CCNI2	0.927411	1.548036	0.739159	0.016498
MOGAT2	1.634649	0.878276	-0.89624	0.037234
FOXO6	3.343491	5.284993	0.660546	0.039765
CYGB	8.78597	5.81381	-0.59572	0.000506
HSPB8	26.46713	16.98138	-0.64025	0.00362
CDC42EP5	33.53397	21.81233	-0.62048	0.009472
COL17A1	30.71816	19.78221	-0.63489	0.012243
CPED1	1.084946	0.59392	-0.86928	0.04759
SLC7A8	13.70955	8.929661	-0.6185	0.000895
ACKR1	11.04376	4.732764	-1.22248	0.010785
CMA1	1.406463	0.298478	-2.23637	0.000322
MAP2	1.116242	0.578919	-0.94722	0.048111
S1PR1	5.448363	3.518338	-0.63093	0.03298
CLGN	0.619476	1.068446	0.786393	0.007116
ELN	8.517686	5.173869	-0.71922	0.004581
NRG1	1.176711	0.537253	-1.13109	0.001822
NTRK2	1.054619	0.470316	-1.16502	0.004599
TSPAN7	6.208731	3.457201	-0.84469	0.000351
CPA3	5.898759	2.857678	-1.04557	0.032422
ALDH1L1	5.026095	2.976859	-0.75565	0.021472

SOSTDC1	1.608445	0.625953	-1.36154	0.034469
C16orf54	2.282455	1.0657	-1.09879	0.003542
GNG7	1.109635	0.60753	-0.86906	6.49E-05
ADH1B	1.855274	0.96131	-0.94856	0.014062
IFNG	0.407385	0.83515	1.035644	0.001037
ELOVL3	0.504459	0.884625	0.810329	0.002357
PINLYP	0.877792	0.5187	-0.75898	0.039161
CD1C	2.221411	0.629458	-1.81929	7.40E-05
VCX3A	0.181882	1.597349	3.134601	0.001642
MEF2C	1.742931	1.013751	-0.78181	0.023337
TM4SF19	0.728217	1.163957	0.676597	0.016498
MYH11	41.53741	20.12587	-1.04536	0.037067
GPRC5D	0.898364	1.399766	0.639813	0.004884
THSD7A	0.647106	0.426477	-0.60153	0.021205
ABCC2	1.384927	0.248434	-2.47888	0.003932
C16orf45	4.425396	2.711921	-0.70649	8.00E-05
CXCL11	8.518708	19.09705	1.164643	0.006804
VSTM4	1.414641	0.933895	-0.5991	0.003301
GBP5	3.387388	7.378603	1.123175	0.008668
OR2B6	0.311138	0.833246	1.421187	2.01E-07
SLC28A3	1.234824	2.008081	0.701512	0.046265
CP	9.124039	5.749548	-0.66622	0.01786
HIST1H4I	7.962797	12.71619	0.67532	0.000106
FAM107A	1.324182	0.650618	-1.02522	0.007642
CXCL10	38.60238	74.63331	0.95113	0.001207
MAGEA9B	0.883732	1.581911	0.839987	0.007758
CACNA2D3	0.685101	0.452544	-0.59826	0.006156
LAMA2	1.977508	1.302038	-0.60291	0.049328
CASQ2	2.848924	1.41467	-1.00995	0.026312
FCER1A	1.863532	0.731214	-1.34967	1.41E-06
FAM171A2	1.432393	2.34897	0.7136	0.010218
ADAM23	1.86646	0.994622	-0.90808	0.013107
MMP10	18.86935	7.170235	-1.39595	0.000848
CCNE1	9.243101	14.03185	0.602256	0.000332
ADRA2A	1.51648	0.977146	-0.63408	0.010445
PDE3A	0.646838	0.407454	-0.66677	0.01166
HOXD4	0.957381	0.635653	-0.59086	0.016802
SPOCK3	2.129469	0.970451	-1.13377	0.026964
ABCC3	13.12163	8.344578	-0.65304	0.004384
MAGEA12	3.331234	6.334554	0.927186	0.036207
SNAI3	1.178609	0.597154	-0.98091	0.026253
MAGEA3	8.868196	14.14748	0.673832	0.002381
WNT5A	16.92906	10.59403	-0.67625	0.035458
SOX17	1.341579	0.878473	-0.61086	0.025987

MAP1B	3.856378	2.30574	-0.74202	0.022601
PACRG	0.96625	0.473447	-1.02919	0.011759
SELENOP	8.161003	5.21991	-0.64472	0.010153
TMSB15A	2.745851	6.452932	1.232701	0.016676
CXCL14	59.87204	24.76875	-1.27336	0.000307
CGNL1	1.086093	0.692399	-0.64947	0.019563
THBS1	46.48395	29.89226	-0.63696	0.046555
CYTL1	2.828159	1.058418	-1.41795	0.022555
HTR7	1.793151	1.046849	-0.77644	0.006365
MMRN1	0.725174	0.337628	-1.10289	0.003094
TMEM220	0.682925	0.440222	-0.63349	0.005134
RDM1	1.063292	1.863493	0.809472	0.000115
EDNRB	3.051589	1.714172	-0.83205	0.030017
TNS1	9.291636	5.627315	-0.72349	0.017944
SYNPO2	6.661719	3.132671	-1.0885	0.015004
ZNF671	1.16291	0.750275	-0.63225	0.001334
CAVIN2	4.488623	2.910004	-0.62525	0.019154
E2F1	12.24664	18.60246	0.603107	1.81E-05
ACTL8	0.510882	0.844526	0.72515	0.01951
CRH	14.79944	54.01292	1.867762	0.0244
PLAC9	3.907627	2.489198	-0.65061	0.00696
NGFR	3.549043	1.891124	-0.90819	0.001666
IGF2	461.7419	258.8101	-0.83519	0.002732
MYO3B	0.756815	0.421191	-0.84547	0.015004
ADAMTS15	1.625573	1.048707	-0.63234	0.033858
AKR1C1	16.59985	8.055186	-1.04318	0.015055
APBB1IP	3.043914	5.261509	0.789549	0.01671
IGFBP6	38.8597	23.87035	-0.70306	0.039043
C16orf89	1.050406	0.351434	-1.57962	0.003099
CLDN3	15.01873	37.3997	1.316264	0.016119
MAST1	0.410903	0.671866	0.709376	0.001453
CTSG	2.996636	0.745832	-2.00642	0.001097
METTL7B	2.736321	4.405698	0.687133	0.049823
TEK	1.505333	0.91768	-0.71402	0.00802
NEFM	0.950205	0.471643	-1.01054	0.031893
FOSB	27.66377	16.94688	-0.70698	0.029374
GBP1	17.18401	27.2882	0.667211	0.02562
LYNX1	5.146772	2.944464	-0.80566	0.034637
PDGFRA	3.431797	2.050666	-0.74287	0.024035
CLU	86.94141	48.23621	-0.84993	0.008069
ABCG2	2.926906	1.067498	-1.45514	0.042209
UGT1A8	3.53913	1.464423	-1.27306	0.000875
CACNA1C	0.685511	0.436768	-0.65031	0.004779
CDKN2B	7.617412	12.05475	0.662229	0.038347

ZEB1	2.685235	1.657143	-0.69635	0.04759
NSG1	7.090262	3.74561	-0.92064	0.007681
ZFYVE28	1.117159	0.743153	-0.5881	0.025656
ADAMTS9	1.520165	0.843416	-0.84991	0.009943
PTN	33.99921	55.25272	0.700544	0.007681
RASL12	4.226892	2.475999	-0.77159	0.037626
FAXDC2	1.158758	0.717217	-0.6921	0.000945
CLEC2L	0.506645	1.178544	1.217959	0.002257
CES1	41.4247	20.28401	-1.03015	0.024149
ITIH5	1.894436	1.107935	-0.7739	0.001094
E2F3	7.276104	13.35864	0.876535	0.00183
CCL5	36.25385	61.16153	0.75449	0.002122
KRT9	0.46748	0.92746	0.988381	0.022065
LIMS2	3.321443	2.090518	-0.66795	0.045646
TPBGL	0.502119	0.882971	0.814337	0.020024
LAMC3	2.240668	1.461203	-0.61677	0.001928
MARCO	3.547852	6.711542	0.919698	0.014613
AKR1B10	65.61197	36.29327	-0.85426	0.038964
TSPAN18	2.861732	1.790019	-0.67691	0.013379
ASCL1	0.669344	2.166522	1.694561	0.004712
ADAM28	1.547083	1.000436	-0.62892	0.00696
ARHGAP6	1.137536	0.74085	-0.61866	0.001212
ARHGEF6	3.844684	2.433084	-0.66008	0.007269
ANGPTL1	1.330987	0.867964	-0.61679	0.023968
ITGA8	1.464702	0.803194	-0.86679	0.004372
APOC1	29.06706	45.88424	0.658614	0.000351
EMCN	2.10067	1.181001	-0.83084	0.006577
C1QTNF3	2.746007	1.569587	-0.80695	0.002971
PODXL2	20.16626	31.66985	0.651167	0.004437
HOXD1	0.656691	0.409674	-0.68074	0.049328
SLIT3	3.107249	1.712325	-0.85968	7.39E-05
PEG3	0.947846	0.282221	-1.74783	0.003478
ACP5	16.72045	32.1992	0.945411	0.045198
UGT8	0.894503	1.622078	0.858685	0.022671
XCL1	1.640135	2.46774	0.589376	0.005426
IGFBP2	23.42443	13.29413	-0.81722	0.003595
RHEX	3.926295	2.342925	-0.74486	0.011999
PLCG2	2.725773	1.459727	-0.90097	0.000381
NEFL	3.893818	0.45566	-3.09516	0.001969
MROH2A	2.61436	1.48206	-0.81885	0.040359
PRSS3	5.131982	9.094026	0.825403	0.006378
PLAC8L1	0.396827	0.609618	0.619394	0.029872
TCF21	0.926302	0.536664	-0.78747	0.001817
CDH12	0.317074	0.698512	1.139465	0.0353

BAALC	2.417081	1.594991	-0.59972	0.014485
GMFG	17.23147	11.08674	-0.63621	0.019887
KCNK13	0.735403	1.557426	1.082557	0.019339
